# Sudden visual loss in the untreated eye of a patient with neovascular glaucoma following an intravitreal bevacizumab injection: A case report

**DOI:** 10.3892/ol.2013.1382

**Published:** 2013-06-07

**Authors:** DING XU, LIUMEI HU, BING WANG, FANG WANG

**Affiliations:** Department of Ophthalmology, Shanghai 10th People’s Hospital, Tongji University School of Medicine, Shanghai 200072, P.R. China

**Keywords:** anti-vascular endothelial growth factor, neovascular glaucoma, bevacizumab, anterior ischemic optic neuropathy

## Abstract

The current study presents the case of a patient with a rare adverse event characterized by sudden vision loss in the untreated eye following an intravitreal injection of bevacizumab for neovascular glaucoma (NVG). The patient was diagnosed with NVG refractory to Ahmed glaucoma valve implantation and a vitreous hemorrhage in the right eye, which was treated with 1.25 mg intravitreal bevacizumab. Ten days after the bevacizumab injection, the left eye exhibited sudden visual loss. The patient’s best-corrected visual acuity (BCVA) decreased from 80 to 25 letters [Early Treatment Diabetic Retinopathy Study (ETDRS) chart]. A fundus examination revealed a swollen optic disk with unclear boundaries, retinal hemorrhages and thinning retinal vessels. Fundus fluorescein angiography (FA) identified hyperfluorescence in the optic disk and an enlarged foveal avascular zone. The visual field revealed quadrantal defects that confirmed the diagnosis of anterior ischemic optic neuropathy associated with ischemic maculopathy. Six months later, following medical treatment, the patient’s BCVA was increased to 44 letters. However, a clinical examination found neovessels with one papilla disk (PD) above the disk. Laser photocoagulation treatment was administered immediately. The area of neovessels above the disk was reduced to 1/4 PD at the last follow-up. In conclusion, although intravitreal anti-vascular endothelial growth factor (Bevacizumab) is an effective treatment for neovascular ocular diseases, its adverse effects must be taken into consideration for the treatment of NVG. Photocoagulation remains an effective treatment for proliferative diabetic retinopathy.

## Introduction

Bevacizumab (Avastin) is a full-length humanized murine anti-vascular endothelial growth factor (anti-VEGF) monoclonal antibody with a molecular mass of 149 kDa, which binds all isoforms of VEGF (1,2). Bevacizumab functions by inactivating VEGF, thereby inhibiting endothelial cell activation and proliferation (3). At present, the drug is used off-label for the intravitreal treatment of several neovascular and exudative ocular diseases (4). As VEGF is also important for normal physiological processes, including cardiac development (5), maintenance of the microvasculature in a number of organs (6), neural cell survival (7), vasodilation (8), trophic support of the choriocapillaris (9) and endothelial cell recruitment (10), inhibition is also associated with a toxic effect in normal issues. Therefore, the adverse effects of an intravitreal injection of bevacizumab may occur due to the injection or be drug-related (11–13). Drug-related adverse events include inflammation, cataract progression, acute vision loss, central retinal artery occlusion, anterior ischemic optic neuropathy (AION), increased blood pressure, deep venous thrombosis and transient ischemic attack. To date, ischemic retinal and choriocapillaris changes following the use of intravitreal anti-VEGF drugs have received a considerable amount of attention (14,15). However, adverse effects in the untreated eye following intravitreal bevacizumab injection have not been reported. The current study presents a clinical case of sudden vision loss in the untreated eye occurring 10 days after intravitreal bevacizumab treatment for neovascular glaucoma (NVG).

## Case report

### Clinical presentation

**Figure 1.**
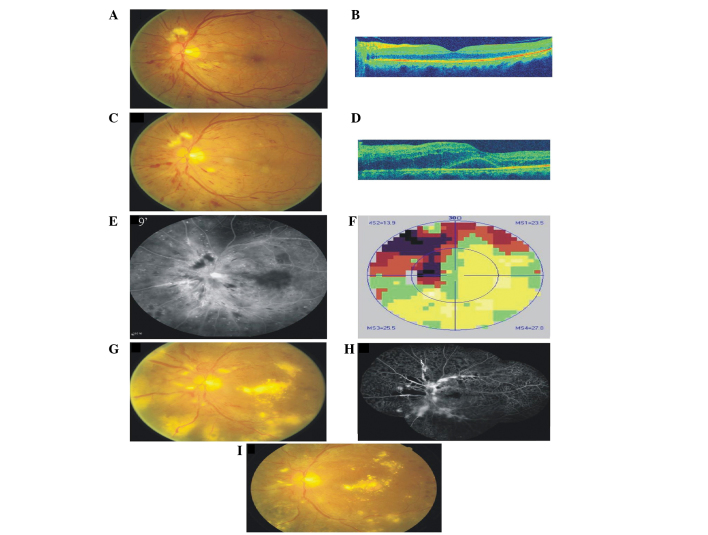
Color, OCT, FA and VF images of the left eye. (A and B) One month prior to administration of intravitreal bevacizumab; (C–F) 10 days post-injection; (G–I) color images and FA of the left eye 6 months later. (A) Color image revealing a clear boundary of the disk, a small cottonwool patch above the disk and a radial hemorrhage at the posterior pole. (B) OCT revealing an almost normal macula profile. (C) Color image revealing a swollen optic disk with unclear boundaries, several retinal hemorrhages and thinning retinal vessels. (D) OCT revealing macular neurosensory retinal detachment. The CRT was 410 *μ*m. (E) FA revealing hyperfluorescence in the optic disk and enlargement of the foveal avascular zone. (F) VF images revealing a quadrantal defect connected with a physiological blind spot. (G) Neovessels of 1 PD area above the disk, vascular ectasia and considerable levels of exudation are present at the posterior pole. (H) FA revealing hyperfluorescence on the disk and superotemporal and inferior areas. There was no non-perfusion in the nasal area. (I) Color images of the left eye 7 months later. The area of neovessels above the disk was reduced to 1/4 PD, vascular ectasia was observed and the level of exudation was reduced. CRT, central retinal thickness; OCT, optical coherence tomography; FA, fluorescein angiography; VF, visual field; PD, papilla disk.

A 47-year-old male presented with severe ophthalmalgia and vision loss in the right eye. The patient had been diagnosed with proliferative diabetic retinopathy (PDR) 1 year earlier. The patient provided written informed consent. The patient underwent pars plana vitrectomy combined with retinal photocoagulation and pan retinal photocoagulation in the right and left eyes, respectively. The individual had also undergone Ahmed glaucoma valve implantation 8 months earlier due to NVG. A general history revealed that diabetes mellitus type 2 had been diagnosed 10 years previously and that the patient was treated with subcutaneous insulin injections. The patient’s best-corrected visual acuity (BCVA) was 20 letters [Early Treatment Diabetic Retinopathy Study (ETDRS) chart] in the right eye and 80 letters in the left eye. The intraocular pressure (IOP) was 50 mmHg in the right eye and 14 mmHg in the left. A slit-lamp examination of the anterior segment revealed corneal edema, rubeosis of the iris, a dilated pupil, the loss of the light reflex, lens opacity and a vitreous hemorrhage in the right eye. A normal anterior segment was observed in the left eye. The retina of the right eye was invisible. A clinical examination of the retina of the left eye revealed a small cotton-wool patch above the disk, hemorrhagic foci at the posterior pole and a laser spot on the peripheral retina (Fig. 1A). Optical coherence tomography (OCT) found an almost normally shaped macula in the left eye (Fig. 1B). The patient was administered with an intravitreal injection of bevacizumab (1.25 mg; Genentech/Roche, Basel, Switzerland) in the right eye. Ten days after the injection, the patient presented with sudden visual loss in the left eye. The patient’s BCVA was now 22 letters in the right eye and 25 letters in the left eye. The IOP was 30 mmHg in the right eye and 14mm Hg in the left eye. The rubeosis of the iris had disappeared and the vitreous hemorrhage was slightly improved. However, the retina was still invisible in the right eye. A biomicroscopic examination of the left eye revealed a swollen optic disk with unclear boundaries, several retinal hemorrhages and thinning retinal vessels (Fig. 1C). The central retinal thickness measured upon OCT examination was 410 *μ*m (Fig. 1D). Fluorescein angiography (FA) revealed delayed arterial filling with hyperfluorescence in the optical disk and an enlargement of the foveal avascular zone (Fig. 1E). The visual field (VF; Fig. 1F) was identified to exhibit a quadrantal defect associated with a blind spot. These symptoms were consistent with a diagnosis of AION associated with ischemic maculopathy.

### Treatment

Compound anisodine was injected around the superficial temporal artery, using 2 ml each time, 10 times in total, and methylprednisolone (20 mg) was periorbitally injected once. Six months later, the patient’s BCVA had improved to 44 letters in the left eye. A clinical examination identified neovessels of 1 papilla disk (PD) area above the disk, the caliber of the vein was different and considerable exudation was observed at the posterior pole (Fig. 1G and H). Laser photocoagulation treatment was administered immediately. The time of exposure was 0.15 sec, the diameter of the spot was 300–500*μ*m, the energy was 340 mW and the number of spots was 1,244. At the last check-up, the patient’s BCVA was 44 letters, the area of neovessels above the disk was reduced to 1/4 PD, vascular ectasia was observed and the level of exudation was reduced (Fig. 1I).

## Discussion

A growing number of neovascular ocular diseases are currently being treated with bevacizumab and side effects are reported frequently. In the current case study, we hypothesized that acute ischemia occurred in the patient’s left eye as a result of the intravitreal administration of bevacizumab in the right eye.

The intravitreal half-life of bevacizumab in the eyes is ∼4.3 days and has been detected in the serum at low concentrations in rabbits (16). The Fc receptor (FcRn) of the antibody is able to cross the blood-retina barrier (17), modulating IgG transport and protecting against its catabolism, thereby leading to a longer serum half-life.

VEGF is a master regulator of angiogenesis. Sufficient concentrations of VEGF must be maintained in the eye to sustain normal functions. Retinal pigment epithelium (RPE)-secreted VEGF has been identified to be critical for ocular development and plays a prominent role in maintaining the choriocapillaris (18,19). Chronic VEGF inhibition leads to choriocapillary dysfunction and results in macular ischemia.

Sinapis *et al* (20) previously reported that the maximum concentration of intravitreal bevacizumab (1.25 mg/0.05 ml) in the injected eye occurred at day 1, whereas in the untreated eye and the serum, the maximum concentration was identified at day 8. In the present study, we hypothesized that the sudden loss of vision in the untreated eye at 10 days post-injection resulted from the gradual distribution of intravitreal bevacizumab from the right eye to the left. This may be through the use of the blood circulation, and may lead to retinal ischemia, particularly macular ischemia. The disappearance of the iris rubeosis and partially dissolved vitreous hemorrhage in the injected eye demonstrates the efficacy of bevacizumab. Whether the retinal ischemia in the injected eye improved following treatment remains unknown as the retina was invisible. In the present case, FA and VF examinations revealed acute retinal ischemia. Further investigation is required to understand the adverse effects associated with using low concentrations of bevacizumab. As the retinal ischemia has not improved and the vessels are under the influence of diatetes mellitus, the neovessels appear above the disk. Although the laser treatment was efficient for the neovessels, further observation is still required. The present case study demonstrates that intravitreal bevacizumab is an effective treatment for neovascular ocular diseases, however, it is also associated with adverse events that must be taken into consideration, particularly as bevacizumab is an off-label treatment. Photocoagulation remains an effective treatment for PDR.
